# A Protocol for the Automatic Construction of Highly Curated Genome-Scale Models of Human Metabolism

**DOI:** 10.3390/bioengineering10050576

**Published:** 2023-05-10

**Authors:** Igor Marin de Mas, Helena Herand, Jorge Carrasco, Lars K. Nielsen, Pär I. Johansson

**Affiliations:** 1 Novo Nordisk Foundation Center for Biosustainability, Danish Technical University, 2800 Lyngby, Denmark; 2 CAG Center for Endotheliomics, Copenhagen University Hospital, 2100 Copenhagen, Denmark; 3 Department of Clinical Medicine, University of Copenhagen, 1165 Copenhagen, Denmark; 4 Australian Institute for Bioengineering and Nanotechnology (AIBN), The University of Queensland, Brisbane 4072, Australia

**Keywords:** genome-scale metabolic model, human metabolism, model construction, constraints-based modeling

## Abstract

Genome-scale metabolic models (GEMs) have emerged as a tool to understand human metabolism from a holistic perspective with high relevance in the study of many diseases and in the metabolic engineering of human cell lines. GEM building relies on either automated processes that lack manual refinement and result in inaccurate models or manual curation, which is a time-consuming process that limits the continuous update of reliable GEMs. Here, we present a novel algorithm-aided protocol that overcomes these limitations and facilitates the continuous updating of highly curated GEMs. The algorithm enables the automatic curation and/or expansion of existing GEMs or generates a highly curated metabolic network based on current information retrieved from multiple databases in real time. This tool was applied to the latest reconstruction of human metabolism (Human1), generating a series of the human GEMs that improve and expand the reference model and generating the most extensive and comprehensive general reconstruction of human metabolism to date. The tool presented here goes beyond the current state of the art and paves the way for the automatic reconstruction of a highly curated, up-to-date GEM with high potential in computational biology as well as in multiple fields of biological science where metabolism is relevant.

## 1. Introduction

Human metabolism is an integral part of cellular function, and many health conditions may be associated with abnormal metabolic states [1,2]. Several of these conditions can be diagnosed by screening for metabolite biomarkers in patient blood or urine [3], and metabolic processes can be used as targets in disease treatment [4,5].

Despite the importance of metabolism and advances allowing for the simultaneous measurement of thousands of metabolites [6], understanding metabolism in a holistic manner in human cells remains challenging. One reason for this difficulty is that the defining feature of metabolism is not the concentrations of bio-molecules themselves (such as metabolites, mRNA or proteins), but metabolic fluxes through reactions, for which concentrations can only be used as indirect proxies for biological activity [7]. This challenge has been addressed by building genome-scale metabolic models (GEMs).

GEMs encompass the metabolic reactions encoded by an organism’s genome [8]. These systems biology tools are built based on the literature and databases and enable the mathematical representation of bio-transformations and metabolic processes occurring within the organism, offering an appropriate framework to integrate the increasing amount of omics data generated by different high-throughput technologies.

GEM development has been initiated for bacteria [9] and has gradually extended to archaea [10], yeast [11], plants [12,13], mouse [14], and humans [15,16]. GEMs have been used for a variety of applications [17], including studies of evolution [18], metabolic engineering [19], genome annotation [20], drug discovery [21] and systems medicine [22], among many others. Reconstructions of human metabolism such as Recon3D [23], HMR2 [24] or the most recent reconstruction of human metabolism, Human1 [25], have been widely used to decipher the mechanism underlying diseases with a strong metabolic component, such as cancer or diabetes, in the context of systems biology, as well as to improve industrial processes involving human cell lines such as Hek293 [26].

The process of constructing GEMs, either from scratch or based on existing reconstructions, is described by Thiele et al. (2013) and Ma et al. (2007) [16,27]. An initial network is usually automatically built from online databases such as the Kyoto Encyclopedia of Genes and Genomes (KEGG), among others. These databases contain information about metabolites, reactions linking them into a network, enzymes catalyzing these reactions and genes coding for the enzymes. The automated approach can obtain the structure of metabolites, the cell compartment they appear in, the stoichiometry of reactions, the structure of enzymatic complexes, etc. At the end of the automated reconstruction, the model is an accumulation of annotations from the chosen databases (and/or from previous reconstructions). Data are merged after identification of entries from different sources corresponding to the same metabolite, reaction or gene. This avoids duplicates, and sometimes allows the completion of missing information in one database using the corresponding entry from another. However, gaps typically remain, and errors persist, which are addressed through thorough manual curation.

As the manual reconstruction of genome-scale networks is laborious and time consuming, many automated network reconstruction tools have been developed to accelerate the reconstruction process, including ModelSeed [28], CarveMe [29], RAVEN [30,31] and kbase [32]. However, most of these reconstructions lack manual refinement, which may result in an inaccurate description of the organism and unreliable predictions of the model [33]. Thus, these automatically generated GEMs must be revised by an expert, which is time consuming and is subject to the biases and errors associated with manual curation, which limits the continuous updating of highly curated and reliable GEMs.

In order to overcome this difficulty, here, we present an algorithm-aided protocol for the automatic construction of highly curated GEMs. The tool is available in a GitHub repository (https://github.com/biosustain/THG, accessed on 25 February 2023) and can be used either to curate and expand existing reference GEMs or to generate metabolic network models from scratch based on the data available in multiple databases in real time.

**Figure 1:**
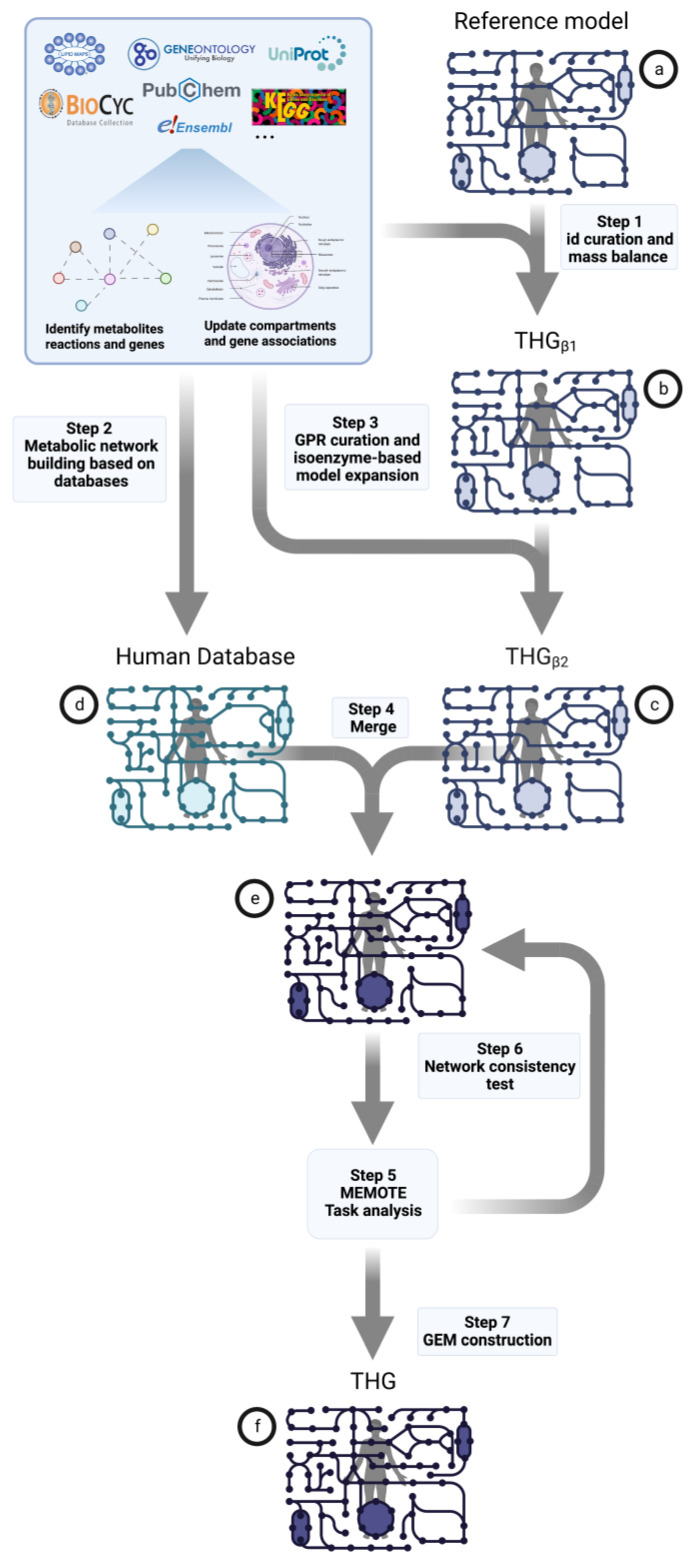
A simplified illustration of the steps involved in the construction of the human GEM (THG) from online databases (KEGG, UniProt, Biocyc, Gene Ontology, etc.) and reference model (**a**). Step 1: i. Automatic similarity-based algorithm for the identification of metabolites. The metabolites are identified based on the name and formula in the PubChem database. Once identified, the annotation of the metabolite is corrected or improved. ii. The metabolic reactions are mass balanced (**b**. THG_β1_). Step 2: Automatic construction of metabolic networks from online databases. In the process, the components of the model (metabolites, reactions, genes) are identified, the reactions are mass balanced, the subcellular compartment is identified and the gene reaction associations are determined (**d**. Human Database). Step 3: Gene reaction protein (GPR) curation and isoenzyme-based model expansion. The subcellular location(s) and GPR(s) are identified using the EC number(s) specified in the reference model as input. If the reaction in the compartment does not exist in the reference model, the reaction is added. For existing reactions, the GPR can be corrected (**c**. THG_β2_). Step 4: Merging of Human Database and THG_β2_ (**e**). Common metabolites, reactions and genes are identified based on the component’s annotation, as well as the substrates and products involved in the metabolic reactions. Step 5: Model assessment with MEMOTE (metabolic model testing). The model’s annotation and consistency are evaluated, as well as the model’s capability to perform essential metabolic functions for cell viability. Step 6: Network consistency test. A network consistency analysis is performed to minimize stoichiometric inconsistency. Step 7: Final constructed model (**f**).

One of the most recent reconstructions of human metabolism (Human1) was used as reference model (Figure 1a) to apply our protocol and generate The Human GEM (THG) (Figure 1f), which, to the best of our knowledge, represents the most extensive and comprehensive reconstruction of human metabolism to date. In this process, a series of intermediate THGs were generated, each improving the reference model at different levels. This series included two intermediate THGs: THG_β1_ (Figure 1b, which checks and corrects the mass balance consistency and improves annotation of the different moieties) and THG_β2_ (Figure 1c, which corrects gene-protein-reaction association, identifies isoenzyme activities and expands the model accordingly), as well as a “Human Database” (Figure 1d) gathering all the known information of the different components associated with cell metabolism in humans. Finally, a THG model was constructed by combining THG_β2_ and the “Human Database” (Figure 1e,f).

The whole process was performed automatically using a set of in-house-developed algorithms designed for this purpose. In brief, the algorithms improved, corrected and expanded metabolites, reactions, proteins and genes annotation, detected and eliminated duplicated entities within the model and mass balanced all metabolic reactions even for large molecules such as glycans in the reference model. Next, the reference model was expanded by incorporating missing information about the genes, metabolites, reactions, proteins and compartments which are currently annotated in many databases following the above-mentioned criteria. In addition, this new approach provided GEMs with new features, such as the use of stoichiometric gene-protein-reaction associations (S-GPRs) [34] and a more accurate formulation for glycans describing their building block and enabling the balancing of glycan-associated reactions.

In addition, since the overall method relies on the information stored in many databases at the moment the model is built, this tool can readily be used to update already existing GEMs as well as provide an excellent platform to generate highly customized GEMs without relying on third-party repositories and when or how these are updated. The tool developed in this work represents a significant advancement in large-scale metabolic network model building and curation. It offers a highly specific and curated approach to automatically reconstruct up-to-date GEMs, which has the potential to have a significant impact not only in the computational biology field but also in all fields of biological science where metabolism plays a critical role.

## 2. Materials and Methods

### 2.1. Metabolic Model Building Pipeline

In this study, we introduce a novel pipeline that employs various algorithms to curate and extend a reference genome-scale metabolic model (GEM) or generate a new model from scratch by gathering relevant information from multiple online databases. The resulting model is provided in the widely used Systems Biology Markup Language (SBML) format, ensuring compatibility with the COBRA toolbox [35,36]. Our pipeline places a strong emphasis on model curation, which is achieved through a series of algorithmic steps: (i) “getGPR” builds and curates gene-protein-reaction associations, (ii) “getLocation” identifies the cellular location of metabolic reactions listed in databases, (iii) “generate_met_annotation” identifies metabolites, (iv) “execute_jaccard” identifies metabolic reactions, (v) “mass balance” balances metabolic reactions and (vi) “test_stoichiometric_consistency” ensures metabolic network consistency. These algorithms are implemented throughout the entire process, which can be divided into three main steps:

- Reference model curation, correction and enrichment (Figure 1a) at the level of reactions, metabolites, genes, gene-protein-reaction association and cellular compartment (Figure 1 step 1 and step 3);
- Database construction from all common online sources of molecular information concerning human metabolism (metabolites, reactions, enzymes, genes, organelles, etc.) (Figure 1 step 2);
- Final model generation from merging human database and curated reference model (Figure 1 steps 4 to 7).

#### 2.1.1. Reference Model Curation 

Firstly, the reference model is curated at different levels by enriching/correcting the annotation, as well as ensuring mass balance. This enriched model enables a broader and more precise integration of high-throughput omics data, while a fully mass-balanced model provides more reliable representation of metabolic flux profiles. Below, this process is explained in more detail.

Metabolites: Metabolites are first identified based on a similarity analysis (“identify_metabolites function”) comparing metabolite names and formula in the model with entries in the PubChem database [37,38]. For each metabolite in the reference model, the algorithm compares its name with the names and synonyms of all the metabolites stored in the PubChem database and determines the longest common sub-string (LCS) for each comparison. 

The LCS analysis provides a general score between the *i*^th^ metabolite in the reference model and each metabolite in the database with values between 0 and 1. The match with the highest score is selected to identify the *i*^th^ metabolite if it is above a given threshold; otherwise, the metabolite remains unidentified. Using LCS as a method to identify metabolites enables the identification of metabolites by name even while minimizing the error due to typos in metabolite names or if the model uses a non-official name or synonym. In this study, different thresholds are tested to determine the robustness of this method.

Next, the same process is carried out for the molecular formula. Here, to account for possible discrepancies due to differences in the metabolite charge between the reference model and databases, the molecular formula is changed to its neutral form for the analysis. Thus, the LCS analysis enables metabolite identification by molecular formula even when radicals, functional groups or molecular structure descriptions differ between the reference model and database.

Once the metabolites have been identified based on the name and formula, the metabolites’ external database IDs are either corrected in the case of incorrect annotation, enriched with a new ID from non-annotated databases and/or added in cases where the metabolite had no external database ID in the reference model.

Reaction: Reactions are curated at different levels.

i. First, reactions are identified by their unique combination of substrates and products. This task is performed using Jaccard index (*JI*) metrics. In this task, KEGG, ChEBI, HMDB and LipidMaps identifiers, as well as the names and formula of the metabolites involved in the reaction, are used as a unique fingerprint that identifies the reaction in the reference model with the information retrieved from online databases.

(1) $${JI}_{T_{i}}={JI}_{S_{i}}+{JI}_{P_{i}}$$

(2) $${JI}_{S_{i}}=\sum_{j=1}^{g}{JI}_{S_{i},S_{j}}+\sum_{j=1}^{g}{JI}_{S_{i},P_{j}}$$

(3) $${JI}_{P_{i}}=\sum_{j=1}^{g}{JI}_{P_{i},S_{j}}+\sum_{j=1}^{g}{JI}_{P_{i},P_{j}}$$

(4) $${JI}_{S_{i},S_{j}}=\frac{S_{i}\cap S_{j}}{S_{i}\cup S_{j}}$$

(5) $${JI}_{S_{i},P_{j}}=\frac{S_{i}\cap P_{j}}{S_{i}\cup P_{j}}$$

(6) $${JI}_{P_{i},S_{j}}=\frac{P_{i}\cap S_{j}}{P_{i}\cup S_{j}}$$

(7) $${JI}_{P_{i},P_{j}}=\frac{P_{i}\cap P_{j}}{P_{i}\cup P_{j}}$$

Here, the algorithm defines *n* groups of substrates (*S*) and products (*P*) for the first set of reactions and g groups of substrates (*S*) and products (*P*) for the second set of reactions, where *n* and g are the number of reactions in the first and second set of reactions, respectively (i.e., reference model and database). The reversibility of each reaction is determined based on information retrieved from online databases. Then, the algorithm compares each *i*^th^ group of substrates and products (from the reference model) with each *j*^th^ group of substrates and products (from the database) and seeks to identify the groups with the highest similarities between sets of reactions (Equations (1)–(3)). Equations (4)–(7) define the *JI* between the groups of substrates and products of the two sets of reactions.

In this study, only perfect matches between reactions and databases are considered. This represents a strong constraint that can, however, be relaxed to accept non-perfect matches. Since metabolite charge may vary between different databases and the reference model, protons are not considered in this analysis;

ii. Finally, the metabolic reactions are mass balanced using the “mass_balance” function. This function sequentially applies four different analyses of the molecular formulas of substrates and products until the balance is achieved.
  1. First, the reaction is analyzed to detect mass balance inconsistencies. This is calculated by considering the molecular formula and the stoichiometry of the species. If the reaction is not mass balanced, a second method is applied;
  2. Here, the metabolic reaction is transformed to a matrix form (M) representing the number of each atom that each metabolite in the reaction has, where rows and columns represent metabolites (substrates and products) and atoms, respectively. The coefficients in the M matrix corresponding to products are multiplied by −1, and the system is solved;
  3. If the previous step provides multiple solutions or no solution, the reduced row echelon form of M matrix is solved. If multiple solutions are found, the one describing the lowest overall stoichiometric coefficients is selected;
  4. Finally, if no solution exists for integer numbers using the previous approaches, the reaction mass balance is formalized as a linear programming problem. Here, the stoichiometric coefficients are the variables to determine, and the difference of atoms between the right and left side of the metabolic reaction is minimized (objective function) while constraining the results to values higher than 0.

In the case where the above-mentioned methods are not able to mass balance the reaction, the algorithm adds a new metabolite as substrate and/or product, and the methods for mass balancing is run again in the same order. The added metabolites can be H^+^, H_2_O, K^+^, Na^+^, Ca^2+^, Fe and radicals (R).

The algorithm also enables the mass balance of reactions involving glycans. Here, glycan building blocks are treated as atoms in a regular molecular formula, which enables the use of the above-mentioned pipeline to mass balance reactions involving glycans.

Genes and proteins: Once the metabolic reactions are correctly identified by applying the JI-based algorithm, the associated genes and proteins IDs are corrected or added if necessary.

Gene-protein-reaction associations: Once the metabolic reactions are identified following the above-mentioned approach, the Enzyme Commission (EC) number is corrected if necessary. The EC number is a numerical code consisting of four digits that describes the enzymatic activity of a given protein that catalyzes one or several reactions. Since enzymatic activity is directly linked to specific proteins, the EC number enables identification of the gene/s encoding for the specific protein catalyzing a given reaction. Furthermore, it is possible to define how these genes are associated with the corresponding metabolic reactions. This association can be either formulated using logic “and”/”or” to describe genes encoding for either isoenzymes or complexes, respectively (GPRs), or further enriched with the number of protomers required to generate a catalytically active unit or enzyme (S-GPRs). To incorporate both GPRs and S-GPRs into the GEM reconstruction, an algorithm developed by Marin et al. in 2019 [34] is incorporated and adapted to this protocol. Here, the EC number/s describing each enzymatic reaction in the model is used as input to gather information from the MetaCyc [39,40], Biocyc [39,41], Humancyc [42], Uniprot [43,44] and KEGG [45] databases.

Next, this information is combined and interpreted by using Levenshtein automaton formalism [46] to generate an abstract syntax tree (AST) which is used to build the corresponding S-GPR. Classical GPRs are generated by removing the stoichiometric information from the S-GPRs. Finally, EC numbers, genes, GPRs and S-GPRs are corrected or enriched in the reference model if necessary.

Cell location: EC number annotated in the metabolic reactions in the model defines the activity of a catalytic unit (enzyme or complex) catalyzing a biochemical reaction. These catalytic units can be located in different cell compartments. The algorithm can identify if the enzyme catalyzing a given reaction is in one or more cell compartments, or, in the case of a complex composed of several subunits, the location is identified as the common location/s of all the subunits. This information is determined by combining information from the KEGG [45], Biocyc [39,40] and Uniprot [43,44] databases and standardized by using GO annotations [47,48]. Thus, the reaction location/s is defined as the cellular location/s of the corresponding catalytic unit/s (enzyme or complex).

In case a given reaction can be present in more than one subcellular location (i.e., two isoenzymes catalyzing the same reaction in two different cell compartments), as many copies of the reaction as compartments where the reaction is present are generated, including the corresponding cellular-location-specific substrates and products and the gene-protein-reaction associations.

#### 2.1.2. Database Model of All Human-Specific Metabolic Information

To expand the already improved reference model, a database collecting all the online available molecular information concerning human metabolism (metabolites, reactions, enzymes, genes, organelles, etc.) is built (Figure 1d). Data from a variety of databases such as KEGG [45], Ensembl [49,50], LipidMaps [51,52], NCBI [53], Biocyc [39,41], Humancyc [42], MetaCyc [39,40], PubChem [37,38], Gene Ontology [47,48], Uniprot [43,44], ChEBI [54,55] and HGNC [56] are queried (Figure 1 step 2). 

The database is constructed to retrieve the same type of information that is stored in the reference model in order to optimize the merging step.

The process starts by defining a list of human metabolic pathways. In this work, the human-specific KEGG PATHWAY reference database pathway maps stored as KEGG Markup Language (KGML), along with the human KEGG pathway map, are collected. All the reactions in each pathway are added to the database along with the associated compounds (substrates and products) and EC number/s. Next, IDs from a variety of databases are added to each metabolite (KEGG [45], PubChem [37,38], LipidMaps [51,52], InChI and InChI key [57], ChEBI [54,55]) together with the corresponding molecular formula from ChEBI, KEGG or PubChem.

The EC number is used to infer the gene/s encoding for the enzyme, isoenzyme and/or complex catalyzing the metabolic reactions. In this process, the corresponding gene products are added to the database with the corresponding HGNC [58], Ensembl [49,50] and Entrez IDs. Next, the information about the associated genes retrieved from different databases is combined and interpreted to generate an AST that is used to build the corresponding S-GPRs and GPRs [34]. 

Finally, the cellular compartment/s where a given reaction is located are determined by matching the EC number/s and the associated gene/s with the NCBI [53], Uniprot [43,44] and Gene Ontology (GO) [47,48] databases. This is defined in the database by annotating the associated compounds (substrates and products) to the corresponding compartment. In case the reaction is located in multiple cellular compartments, a copy of the reaction is generated and stored for each compartment. Here, the gene-protein-reaction associations are modified accordingly, i.e., if different isoenzymes are expressed at different cellular locations, the GPR of each copy of the reaction only contains the genes corresponding to that compartment. In addition, new metabolite entities and compartments are added to the database in case they were not previously accounted for. Finally, reactions are mass balanced following the approach applied during reference model curation.

#### 2.1.3. Reference Model and Database Merging

Once completed, the curated and corrected reference model (Figure 1c) and the database (Figure 1d) are merged into a single metabolic network model (Figure 1e and Figure 1 step 4). To avoid duplicate moieties, overlaps between the reference model and the database are identified by the following strategies: i. overlapped metabolites are determined by metabolite ID and name using the text similarity algorithm, ii. the JI-based algorithm is applied to identify overlapping reactions, iii. duplicated genes are identified by their Ensembl ID.

Since the stoichiometric consistency of a metabolic network resulting from the merging of two stoichiometrically consistent GEMs is not guaranteed [59], a network consistency test is iteratively applied in the merging process (Figure 1 steps 5 to 7). This ensures that the resulting GEM is consistent from a stoichiometric point of view.

### 2.2. Reference Model

The pipeline developed in this work was applied to a recent reconstruction of the human metabolism, namely, Human1 (v1.3.x, version released in February 2022, Figure 1a). Human1 was reconstructed by combining HMR2 and Recon3D GEMs, which represent reconstructions from two of the most widely used GEM lineages. The reference model accounts for 13,069 reactions, 8369 metabolites, 3067 genes, 8022 GPRs and 9 compartments [24]. 

### 2.3. Model Quality Assessment

To evaluate the quality of the models constructed, two methods were used:

- Testing metabolic model consistency and annotation by applying the metabolic model test (MEMOTE) pipeline (Figure 1 step 5); MEMOTE is a standardized tool for evaluating GEMs [60] and benchmarks GEMs in different areas. First, an evaluation of the semantic description of the domain-specific model components, such as flux bounds, metabolic formulas and annotation, among others, is performed. Next, the model is benchmarked in four general areas:
  - GEM annotation according to community standards [61];
  - Basic tests check the formal correctness of a model and verify the presence of components such as metabolites, compartments, reactions and genes. These tests also check for metabolite formula and charge information and GPR rules. General quality metrics, such as the degree of metabolic coverage representing the ratio of reactions and genes, are also checked. Since MEMOTE cannot evaluate S-GPRs and building-block-based glycan formulation Glycan, these tests are provided in a separate table;
  - A model is tested for production of biomass precursors in different conditions for biomass consistency, for nonzero growth rate and for direct precursors;
  - Stoichiometric inconsistency, erroneously produced energy metabolites [62] and permanently blocked reactions are identified by MEMOTE. Errors in stoichiometry may result in the production of ATP or redox [63] and are detrimental to the performance of the model when using flux-based analysis [64].
- Task analysis (Figure 1 step 5): A metabolic task is defined as the capability that a given model must have to metabolize one or more metabolic products from a specific source of substrate/s. Analysis of essential metabolic tasks describing essential metabolic functions for cell viability is performed in the different GEMs and compared with the reference model. The essential tasks are described for Human1 [24] and are available in its GitHub repository (https://github.com/SysBioChalmers/Human-GEM/, accessed on 24 February 2023). The analysis is performed as described by Henriksen et al. in 2022 [21] and integrated into MEMOTE.

## 3. Results

### 3.1. JI-Based Algorithm Identifies Metabolic Reactions Based on the Unique Combination of Substrates and Products

A JI-based algorithm was developed to identify metabolic reactions. This method relies on the unique combination of substrates and products as a fingerprint to identify metabolic reactions without the need for annotation. This approach is especially useful for identifying model reactions with no annotation.

A set of 635 unique reactions with EC numbers, including substrates and products from the “Human Database” metabolic network, was used as a testing dataset to evaluate the capability of the algorithm to identify metabolic reactions. Here, the input of the algorithm was the substrate and products that the algorithm uses to predict the EC number that was compared with the reference. 

Based on Equations (1)–(7), the JI can have values between 0 and 2, where 2 represents a perfect match. In this analysis, only perfect matches were considered (JI = 2).

**Figure 2:**
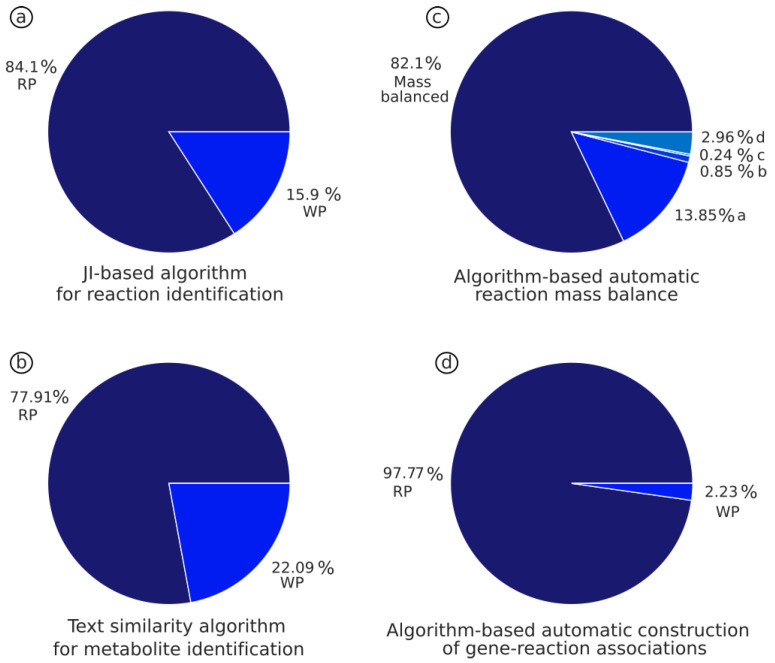
Results of assessment of the different algorithms developed for this protocol. (**a**) Text similarity algorithm to identify metabolites. Graphical representation of the results obtained using a threshold of 0.82 (other thresholds results can be found in Supplementary Material File S1). Dark blue: percentage of right prediction (RP), light blue: percentage of wrong predictions (WP). (**b**) JI-based algorithm to identify metabolic reactions. Dark blue: percentage of right prediction (RP), light blue: percentage of wrong predictions (WP). (**c**) S-GPR building algorithm. Dark blue: percentage of right prediction (RP), light blue: percentage of wrong predictions (WP). (**d**) Algorithm to mass balance metabolic reactions. Dark blue: percentage of reactions mass balanced by the algorithm; the different tones of lighter blue represent cases where the reaction was not mass balanced due to: a. an exchange reaction that is imbalanced by definition, b. a mixture of molecular and glycan building block formulas that cannot be mass balanced, c. at least one of the metabolites having no annotated formula or d. coupled reactions that cannot be mass balanced unless the reactions are decoupled and mass balanced separately.

The algorithm demonstrated a high predictive capability, able to identify up to 84.1% of the metabolic reactions providing the correct EC number (Figure 2a). See Supplementary Material File S1 for more detailed information of the results.

### 3.2. Text Similarity Algorithm to Identify Metabolites by Comparing Names and Formula with PubChem Database

Incorrectly annotated metabolites were identified by applying a novel method that compares metabolite name and molecular formula with the metabolite names (and synonyms) and formulas retrieved from the PubChem database. If the similarity was above a given threshold (between 0 and 1), the metabolites were considered identical, and the PubChem ID was used to identify the metabolite.

Here, a list of 1418 metabolites with the corresponding names, formulas and PubChem IDs extracted from the human database (Figure 1c) were used as a testing dataset. The names and formulas were used as the input of the text similarity algorithm, and the predicted PubChem ID was compared with the annotated one. In addition, to evaluate the robustness of the analysis, different thresholds were tested: 0.1, 0.6, 0.7, 0.8 and 0.82. The percentage of correct predictions as a function of threshold only varied between 77.4% and 77.9%, and the distribution of the results between the different thresholds showed a significant overlap of 99.9% (Figure 2b), which indicates a high accuracy and robustness of the predictions. Detailed results are in Supplementary Material File S1.

### 3.3. Automatic Identification and Rebalancing of Imbalanced Reaction by Using the Mass Balance Reaction Algorithm

Reaction mass balance was performed by the “mass_balance” function. This function uses three different methods that combine linear algebra, matrix calculation and linear programming to ensure that the metabolic reactions are mass balanced. Briefly, the function assesses the mass balance of the reaction, and, if it is imbalanced, the three methods are applied until the reaction is balanced. If the reaction cannot be mass balanced, potential missing species such as protons or water are added, and the different methods are applied again until the reaction is mass balanced. To assess the mass_balance function, a total of 9295 unique reactions from the reference model and the “Human Database” were used as a test dataset. The algorithm was able to mass balance 82.1% of the reactions in the Human1 model. The remaining 17.9% of imbalanced reactions were as follows: i. 77.42% were exchange reactions, which are imbalanced by definition as they only have substrates, ii. 4.75% were a mix of a molecular and glycan formula, iii. 1.32% of the reactions had at least one compound that had no formula and iv. 16.51% of the reactions described coupled reactions that could not be mass balanced unless the reactions were decoupled and mass balanced separately (Figure 2.c). More details can be found in Supplementary Material File S1.

### 3.4. The Gene-Protein-Reaction Algorithm Enables the Automatic Building of GPRs and S-GPR Based on Current Data from Online Databases

GPR building was carried out by the “get_gpr” function. Here, we evaluate the capability of this algorithm to extract information from different databases and build the corresponding S-GPRs. This algorithm uses as input the EC number to identify the associated genes and infers the corresponding S-GPRs and GPRs (for more details see Marin de Mas et al., 2019 [34]). A list of 1074 GPRs with the corresponding EC numbers from Marin de Mas et al., 2019 [34], was used as a testing dataset. Here, the EC number was used as input for the “get_gpr” function, and the predicted GPR was compared with the published one to evaluate the capability of the algorithm to build the gene-protein-reaction associations. The get_gpr function correctly built 97.87% of the GPRs; the remaining 2.23% were partially predicted where one of the isoenzymes in the GPR was missed. This was attributed to the incorrect formatting of the source databases (Figure 2d and Supplementary Material File S1).

### 3.5. Improving and Automatic Curation of the Reference Model Annotation (THG_β1_)

One of the functionalities of the developed protocol is to enrich and curate existing GEMs. The protocol was applied on the reference model by using the “build_model” function. This function incorporates the above-assessed algorithms to evaluate, curate and enrich existing GEMs based on the information currently available in multiple online databases. The databases and identifiers used in the process are KEGG [45], HumanCyc [42], BioCyc [39,41], MetaCyc [39,40], Gene Ontology [47,48], PubChem [37,38], LipidMaps [51,52], Ensembl [49,50], Uniprot [43,44], InChI and InChI key [57], ChEBI [54,55], HGNC [56] and NCBI [53].

**Figure 3:**
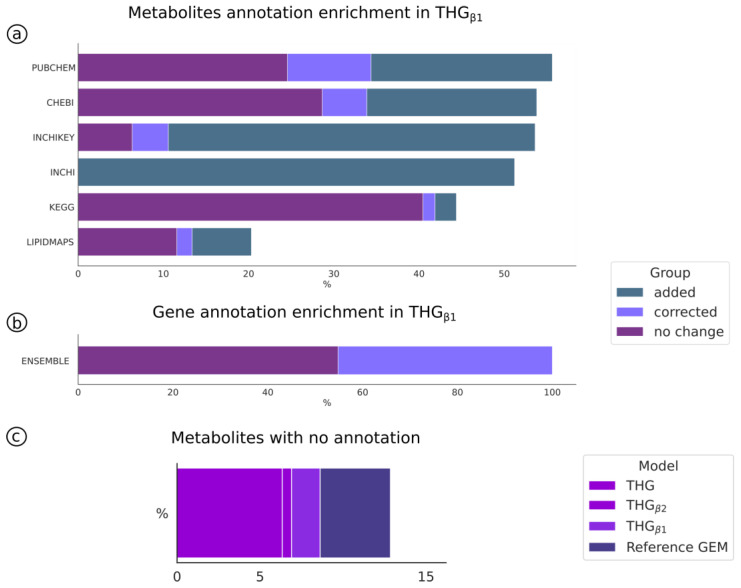
Metabolite and gene annotation enrichment in THG_β1_. (**a**) Percentage of metabolite IDs corrected (lila), added (dark green) in THG_β1_ or right in the reference model (purple). (**b**) Percentage of gene Ensembl IDs corrected or added in THG_β1_. (**c**) Overall percentage of metabolites with no annotation in THG_β1_, THG_β2_ and the reference model.

The resulting model (THG_β1_, Figure 1b) accounted for the same number of elements as the reference model; however, it was enriched in metabolites, genes and reactions IDs, as well as mass balanced. Metabolite annotation was enriched by 4.13%, 26%, 9%, 31%, 53% and 49% for KEGG, ChEBI, LipidMaps, PubChem, InChI and InChI key, respectively (see Figure 3a). In total, the number of unannotated metabolites was reduced by 33% (Figure 3c). All the genes accounted for annotation in the reference model; however, 44% were corrected in THG_β1_ (Figure 3b). The reaction imbalance in the reference model was also assessed. Although the algorithm was able to mass balance reactions with “R” or “X” groups, the reactions with these groups in the reference model were not modified when imbalanced, as the molecular composition of these groups was unclear in each reaction (typically pools in the model). Other reactions that were excluded from the mass balance analysis were: sink, exchange and biomass production reactions. Only one reaction in the reference model had to be rebalanced. More details are in Supplementary Material Files S2 and S3.

### 3.6. Automatic Enrichment and Expansion of the Reference Model by Adding Isoenzyme Reactions (THG_β2_)

**Figure 4:**
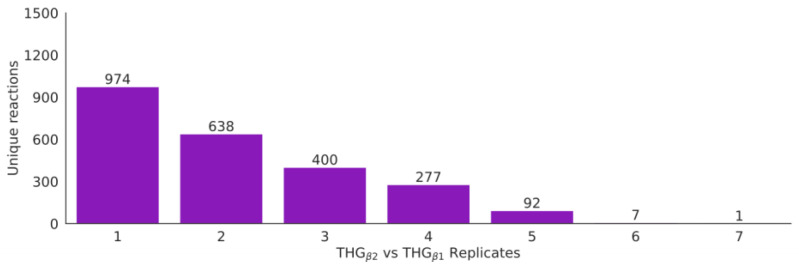
Isoenzyme-based reaction expansion. Detail of the number of reaction and the number of isoenzyme replicates in THG_β2_ compared to in THG_β1_.

**Figure 5:**
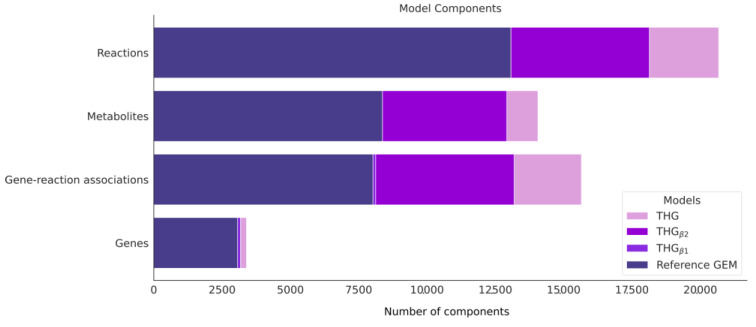
Number of different model components (reactions, metabolites, genes and gene-protein-reaction associations) in the THG model series and the reference model.

THG_β1_ (Figure 1b) was expanded by adding reactions corresponding to isoenzymes with activity in different compartments than the ones originally described in the reference model. This analysis was performed by applying the function “build_model” with extra parameters that enabled the addition of reactions associated with isoenzyme activity and the corresponding metabolites if required. The function uses the EC number of the metabolic reaction in the model to identify the associated genes and then the cellular location where these proteins are located. In the case of complexes, the common location/s of all the subunits defines the subcellular locations. If the reaction is not annotated in one of the inferred compartments, a new reaction is added to the model with the corresponding S-GPR, and new metabolites are added to the compartment if they were not previously annotated. As a result, 5358 new reactions were added describing new isoenzyme activity in different compartments (Figure 4). The final model THG_β2_ (Figure 1c) accounted for 18,136 reactions, 12,919 metabolites, 3174 genes and 13,188 gene-protein-reaction associations (more details in Supplementary Material File S2 and Figure 5).

### 3.7. Applying the Protocol to Build a Database of the Human Metabolism Using Currently Available Online Database Information

The protocol developed in this work also enables the construction of a metabolic network model from scratch by collecting the currently available data stored in online databases (Figure 1 step 2). This was performed by using the function “generate_database”. This function queries the different databases in real time and generates the metabolic network model on the go, which ensures that the generated metabolic network incorporates the most up-to-date information. The input is a list of metabolic pathways, and, in this case, a list of 87 human-specific KEGG pathways was used. It included pathways associated with carbohydrate metabolism (15 pathways), energy metabolism (three pathways), lipid metabolism (15 pathways), glycan metabolism (12 pathways), amino acid metabolism (21 pathways), cofactors and vitamins (12 pathways), terpenoids and poliketides (two pathways), nucleotide metabolism (two pathways), secondary metabolites (two pathways) and xenobiotics biodegradation (three pathways). A more detailed list can be found in Supplementary Material File S2. The resulting metabolic network model accounted for 2930 reactions, 3849 metabolites, 1485 genes and 2838 gene-protein-reaction associations. This analysis was restricted to human-specific reactions only; however, a metabolic network building including general reactions can be performed, generating larger networks. The model in .xml format can be found in Supplementary Material File S2.

### 3.8. Applying the Protocol to Combine THG_β2_ and the Database of Human Metabolism into a Large and Comprehensive Reconstruction of Human Metabolism: THG

Once the database of the human metabolism (Human Database) and the refined and expanded reference model (THG_β2_) were generated, the “merge_metabolic_networks” function was applied (Figure 1 steps 4 to 7) to combine both metabolic networks into the largest and most comprehensive reconstruction of the human metabolism to date (THG) (Figure 1f).

Overlapping metabolites were identified using the developed text similarity function to compare metabolite IDs between the THG_β2_ and Human Database networks. Overlapping reactions were identified by applying the JI-based function to compare the THG_β2_ against the database. Here, 1105 metabolites and 206 reactions were found to be equivalent (overlapping) between the two metabolic networks.

The merged model accounted for 20,677 reactions, 13,840 metabolites, 3392 genes and 15,651 gene-protein-reaction associations, representing an increase of 58.2%, 65.4%, 10.6% and 95.1% in the number of reactions, metabolites, genes and gene-protein-reaction associations, respectively, compared with the reference model (Figure 5 and Supplementary Material File S3).

### 3.9. Model Assessment via MEMOTE and Task Analysis

**Figure 6:**
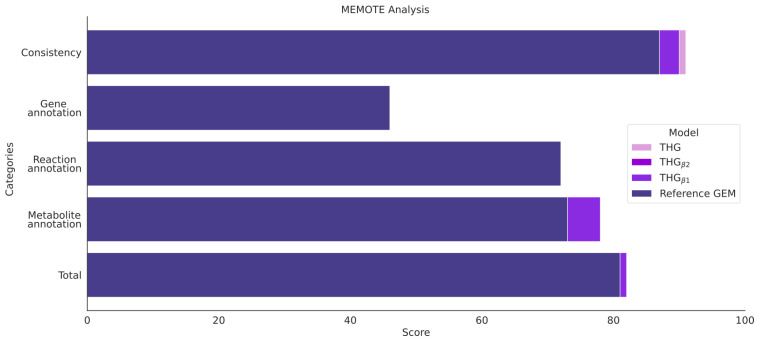
Summary of the scores obtained by the THG model series and the reference model in the different categories of MEMOTE analysis. The scores could have a value between 0 and 100.

The metabolic network models were assessed using MEMOTE, a standardized pipeline for GEM testing (Figure 1 step 7). This pipeline tests the consistency of mass balance, energy and biomass cell functions, as well as the annotation and gap filling of the metabolic network models. The GEMs generated using the developed protocol achieved similar scores to the reference model. Here, THG_β1_ achieved a score of 82, and THG_β2_ and THG achieved 81, while the reference model was scored as 81 (out of 100) (Figure 6). 

All the models showed a similar performance in the consistency analysis; however, THG_β2_ showed a higher percentage of mass- and charge-balanced reactions. This was due to the isoenzyme-based expansion of the model, which duplicated a higher proportion of mass-balanced reactions in the corresponding subcellular locations, increasing the overall percentage of balanced reactions in the model. The TGH model presented a lower percentage of imbalanced reactions, since all the metabolic reactions added from the “Human Database” were mass balanced, thus, diluting the effect of the imbalanced reaction in the reference model. Since the SBML format does not accept glycans formulation using building blocks, this information is stored in the annotations attributed for each metabolite, so it was not considered during the mass balance assessment in MEMOTE.

In the metabolite annotation section, the TGH series showed a clear improvement (score of 78) compared with the reference model (score of 73). Regarding the reactions and gene annotations, all the models showed similar scores (72 and 46 for all the models, respectively). It is worth noting that MEMOTE analysis only evaluated the number of entities with annotation, so all the annotations that were corrected in the process were not considered here. Additionally, relevant IDs that were improved by our protocol, such as LipidMaps for metabolites or Ensembl for genes, were not considered in the analysis.

The “metabolic coverage”, defined as the number of reactions divided by the number of genes, provides a score to evaluate the degree of detail in a model. Here, the score in the reference model was higher than in THG_β1_ (4.26 and 4.12, respectively); this was due to the duplicate genes that were eliminated in THG_β1_ (Supplementary Material File S3). However, since the isoenzyme-based expansion performed to construct THG_β2_ was based on the associated genes, this score increased to 5.71 in this model. This score was even higher in THG (6.11), showing a more complete coverage of the relation between metabolism and gene regulatory mechanisms. A metabolic task analysis was also carried out on the models to determine if they were able to perform the essential metabolic functions necessary for cell viability [21,23]. The task list (see Supplementary Material File S4) was originally developed for the reference model, and the models in the THG series continued to be able to perform all the activities.

## 4. Discussion

In this work, we presented a protocol for the automated curation, expansion and/or building of high-quality genome-scale models of human metabolism. This protocol is available as a GitHub repository, and a more descriptive pseudo-code is available as supplementary material (Supplementary Material File S5). The algorithm was applied on the Human1 GEM [24]. In order to demonstrate the capabilities of the developed protocol, several GEMs were generated: i. THG_β1_ accounted for the same number of entities as the reference model and improved, corrected and enriched the annotation of metabolites, genes and reactions and corrected the unbalanced reactions, ii. THG_β2_ added isoenzyme reactions to other compartments, iii. the Human Database gathered information for metabolites, reactions, genes and S-GPRs of all the human-specific pathways using the same quality criteria as in THG_β1_ and THG_β2_ and iv. THG was the result of combining the THG_β2_ and Human Database network models and represents the most extensive and comprehensive reconstruction of human metabolism to date (Figure 1).

The tool incorporates a novel machine-learning method based on text similarity that was developed to identify incorrectly annotated metabolites in a reference model. This novel method identifies incorrectly annotated metabolites based on their name and molecular formula, as well as corrects and improves annotations for already annotated metabolites. The algorithm was able to reduce the number of incorrectly annotated metabolites by half (from 15% in the reference model to 7% in THG_β1_). The number of metabolites with lipid annotation in the reference model was increased by 50% by adding the LipidMap ID to metabolites missing this identifier. This is especially relevant for integrating the increasing generation of lipidomics datasets [65] into GEM-based analyses. Regarding the other metabolite databases, the algorithm improved the model’s annotation by between 10 and 80% (Figure 3a and Supplementary Material File S3). In addition, an InChI key was added to all the metabolites. The incorporation of InChI identifiers has the potential to enhance the accuracy of the metabolite formula descriptions and, ultimately, improve the effectiveness of the mass balance algorithm.

To identify metabolic reactions, a novel JI-based method was developed. This method uses a reaction’s unique combination of substrates and products as a fingerprint and searches for the most likely matching reaction in the KEGG database [45]. The algorithm was able to identify the reaction ID of 9% of incorrectly annotated reactions, and the annotations of 75 reactions in the reference model were improved (Supplementary Material File S3).

GEMs have become a widely used platform to integrate and analyze multiple omics data. This process is carried out by matching IDs from the high-throughput platforms with the ones annotated in the model. Consequently, improving the annotation of the cellular constituents increases a GEM’s capability to integrate multiple omics data, enhancing the model’s predictive potential.

A pipeline combining four different techniques to mass balance metabolic reactions was also implemented and applied on the reference model. As a result, all the reactions in the reference model were assessed to ensure the mass balance. Metabolic modeling relies on the mass conservation law that stipulates that no mass is created or destroyed in an isolated system. Thus, properly mass-balanced reactions are essential for consistent metabolic modeling analysis, avoiding mass balance inconsistencies, such as ATP-free biomass production, which may seriously compromise the reliability of GEM-based predictions.

In addition, the reference model was expanded in two steps. First, the EC numbers of the metabolic reactions were used to identify isoenzyme activity in other cellular compartments, and reaction metabolites, genes and S-GPRs were added accordingly. Here, 5158 reactions and 3577 metabolites were added to THG_β1_ (Supplementary Material File S3). Next, a metabolic network model of the human metabolism was generated by gathering the online available data from a variety of databases of molecular entities (metabolites, genes, proteins, reactions and compartments). The resulting metabolic network consisted of 2930 reactions, 3849 metabolites, 1485 genes and 2838 gene-protein-reaction associations. This model was built very conservatively, including only human-specific reactions in the “Human Database” (Figure 1 step 2), which limited the extent to which the reference models can be expanded. Less restrictive criteria, including universal reactions, will enable further expansion of current GEMs.

After merging THG_β2_ and the Human Database, the resulting GEM (THG) accounted for 20,677 reactions, 13,840 metabolites, 3394 genes and 15,651 gene protein reactions (Figure 5), representing the most extensive and comprehensive reconstruction of human metabolism to date. This expansion increased the number of reactions and metabolites by 58.2% and 65.4%, respectively, and provides a broader and better overview of the human metabolism, which is pivotal to improve our understanding of the molecular mechanisms underlying a variety of phenotypes.

In order to assess the quality of the models generated by our protocol, MEMOTE and a task analysis defining essential cell tasks were performed on the reference model and the THG series. The models in the THG series showed a similar performance in MEMOTE analysis, with scores between 81 and 82 (over 100) compared to a score of 81 for the reference model. The differences were mainly due metabolite and reaction annotation, as well as network consistency and connectivity (Supplementary Material File S4). All the GEMs were able to carry flux through all the essential tasks.

Thus, the presented protocol substantially expanded and improved one of the latest reconstructions of human metabolism (Human1) i. from an annotation point of view, which increases the model’s omics integration capabilities and ii. in terms of the number of moieties (reactions, metabolites, genes and gene-protein-reaction annotations) while keeping the mass balance and network consistency. The model generated by the presented protocol enables a broader analysis of human metabolism using high-quality and consistent models with improved annotation that allows for more and better integration of omics data, enhancing the model’s predictive capabilities.

## 5. Conclusions

The current work presented a computational tool combining algorithms based on machine learning, data mining and metabolic modeling to robustly curate, correct and expand GEMs. In addition, the tool developed here has the potential to construct highly curated GEMs without the need for a reference model. These novel methodologies were applied to generate an improved and expanded version of one of the latest reconstructions of human cell metabolism (Human1): THG. The MEMOTE pipeline was used to evaluate the new models, and all showed a better or similar performance compared to the reference model. This indicates that the developed protocol is not only able to generate larger models but also high-quality, consistent and robust metabolic network models.

This protocol gathers all the known information about human metabolism retrieved from many databases into a comprehensive model in COBRA format, which bridges the gap between the knowledge stored in databases and COBRA tools for the analysis of metabolic models.

In addition, the computational tool developed in this work has the potential to change the paradigm of how GEMs are built since it establishes the foundations for the automatic generation of high-quality and robust up-to-date GEMs. This enables the fast generation of curated GEMs, gathering the most recently updated molecular information of cell metabolism while providing a standardized pipeline to construct a GEM. 

## Data Availability

The software developed in this study can be found as a GitHub repository at: https://github.com/biosustain/THG, accessed on 24 February 2023.

## Supplementary Materials

The following supporting information can be downloaded at: https://www.mdpi.com/article/10.3390/bioengineering10050576/s1, Supplementary Material File S1: Assessment of the developed algorithms (JI-based reaction identification, text similarity function for metabolite identification, GPR building algorithm and reaction mass balance algorithm); Supplementary Material File S2: THG series and the reference models in SBML format; Supplementary Material File S3: Summary of the comparison between THG series model and the reference model; Supplementary Material File S4: MEMOTE and task analysis of reference and THG series models; Supplementary Material File S5: Supplementary Figure S1 describing protocol structure and pseudo-code.

## Abbreviations

GEM	Genome-scale metabolic model
KEGG	Kyoto Encyclopedia of Genes and Genomes
ChEBI	Chemical Entities of Biological Interest
THG	The human GEM
S-GPR	Stoichiometric gene protein reaction
GPR	Gene reaction protein
MEMOTE	Metabolic model testing
LCS	Longest common sub-string
JI	Jaccard index
EC	Enzyme commission
AST	Abstract syntax tree
KGML	KEGG Markup Language
GO	Gene ontology
