# Supplementary material for: A Protocol for the Automatic Construction of Highly Curated Genome-Scale Models of Human Metabolism
Source: Bioengineering (Basel). 2023 May 10;10(5):576. doi: 10.3390/bioengineering10050576 (PMC10215578; doi:10.3390/bioengineering10050576)
Supplement: Supplementary file 1 [file bioengineering-10-00576-s001.zip › Marin de Mas et al-Supplementary Material file S5.pdf]

1. Graphhical representatioon of the code for the automatomatic construction of GEMs

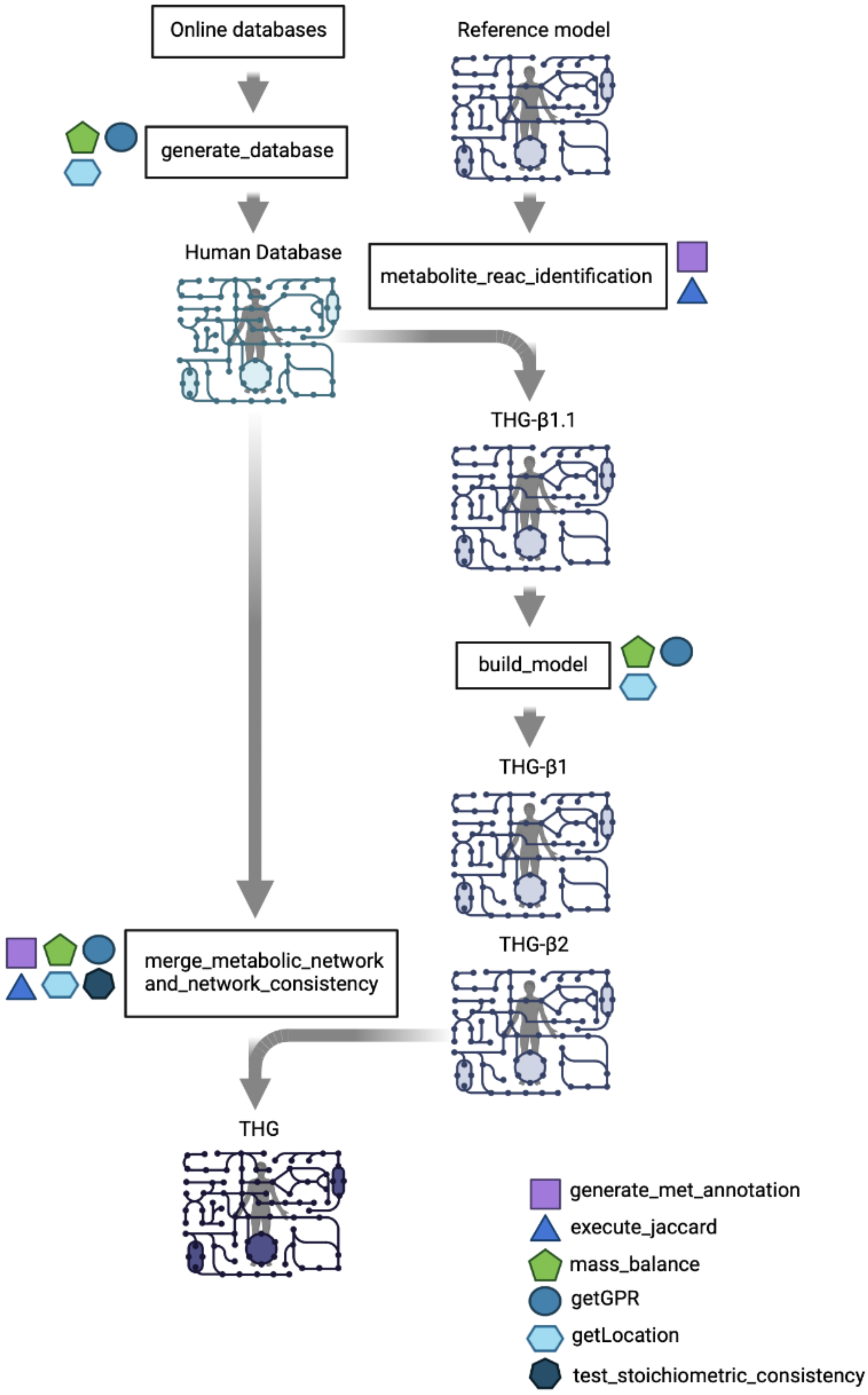

Supplementary figure 1: Figure representing the different steps described in the protocol to construct genome-scale models of human metabolism together with the developed algorihtms to build, curate, test and refine the constructed GEM

2. Pseudo Code for "Building model" process

A. Improve metabolite annotation in the refernce model
# A.1 Gather metabolites info from a `model`, filtering out from `identifiers`.
met_list = empty list
for each met in model.metabolites do the following:

```
    create a tuple met_tuple with elements met.name, met.formula, met.annotation, met.id[:-1]
    if met_tuple is not in met_list then:
        add met_tuple to met_list

# A.2 Identify metabolite annotations .
    unnanotated = []
    annotated = []
    met_annotation = {}
    # Loop through each metabolite in the metabolite list
    for name, formula, annotation, iden in met_list:
        # Identify the metabolite
        result = identify_metabolite(name, formula, iden)
        met = [name, formula, annotation, iden]  #Identify metabolite annotation in different databases
database
        # If the metabolite could not be identified, add it to the list of unannotated metabolites
        if result is None:
            unnanotated.append(met)
        # Otherwise, write the result to output file and add metabolite to the annotated metabolites list
        else:
             met_annotation[met.id] = met #Save the resulting metabolite annotation dictionary in
"met_annotation" variable
            annotated.append(met)


## B. Improve reaction annotation in the refernce model
# B.1 Gather reactions info from a `model`, filtering out from `identifiers`.
result = {}
# Find all reactions in the input model.
for n, reaction in enumerate(model.reactions):
    result[reaction id] = result  + [identify_reaction(reaction, n)]

# B.2 Execute JI on metabolic reactions
met_annotation = {}
for id1, l, r, a, rev in zip(reaction id 1, substrates and products and their stoichiometries in reaction 1):
    for id2, l2, r2, b in zip(reaction id 2, substrates and products and their stoichiometries in reaction 2):
        a, b = str(a), str(b)
        # for reactions "a+b → c+d" and "e+f → g+h", compare the groups [a,b],[e,f] and [c,d],[g,h]
        Jllrr = jaccard(l.split(','), l2.split(',')) + jaccard(r.split(','), r2.split(','))
        if rev == True: # if reaction is reversible
            # for reactions a+b → c+d and e+f → g+h, compare the groups [a,b],[g,h] and [c,d],[e,f]
            Jlrrl = jaccard(l.split(','), r2.split(',')) + jaccard(r.split(','), l2.split(','))
        else:
            Jlrrl = 0
        if Jllrr == 2.0 or Jlrrl == 2.0:
            met_annotation[id1] = [attributes in reaction 1, a,l,r,b,l2,r2,2.0] #Save the resulting reaction
annotation dictionary in "met_annotation" variable
            break

## C. Improve model annotation
```

```
annotate_cobra_model(output_cobra_model_filen_name, met_annotation, reac_annotation)

## D.  mass balance reactions, inprove genes and S-GPR/GPR annotation and isoenzyme-based model
expansion
model = read_sbml_model('models/output_cobra_model_filen_name.xml') # Read in the model from
an SBML file
with model: # Loop over reactions in a model
    for reac_count, x2 in enumerate(model.reactions[0:]):
        ## Collect information from the reaction
        # Get the upper and lower bounds for the reaction
        bounds = x2.bounds
        # Get the EC code for the reaction
        try:
        if 'ec-code' in x2.annotation:
            EC = x2.annotation['ec-code']
        else:
            EC = ''
        # Get the species in the reaction
        species = [s.id for s in x2.reactants] + [s.id for s in x2.products]
        # Get the stoichiometric coefficients for each species
        species3 = [{s.split(' ')[0]: -1} if len(s.split(' ')) == 1 else {s.split(' ')[1]: -abs(float(s.split(' ')[0]))}
for s in re.split(' --> | <=> ', re.sub('[a-z]+', '',x2.reaction))[0].split(' + ')] + [{s.split(' ')[0]: 1} if
len(s.split(' ')) == 1 else {s.split(' ')[1]: float(s.split(' ')[0])} for s in re.split(' --> | <=> ', re.sub('[a-z]+',
'',x2.reaction))[1].split(' + ')]
           species3 = dict(ChainMap(*species3)) # Convert the list of species and coefficients to a
dictionary
        # Get the GPR for the reaction
        gpr = x2.gpr
        # Get the annotations for the reaction
        annotation2 = x2.annotation
        # Get the compartments for each species
        locations = list(set([model2.metabolites.get_by_id(s).compartment for s in species]))

        ## Mass balanced
        mb = x2.check_mass_balance()   # Check if the reaction is mass balanced
        all_met_have_formula_test = min([1 if ListOfMetFrom[x] else 0 for x in species]) # Check if all
metabolites in the reaction have a formula
        # If all metabolites have a formula, replace the metabolite IDs with the corresponding formula
        if all_met_have_formula_test == 1:
            FromList=list()
            for y in species:
                eq = re.sub(y, ListOfMetFrom[y], eq)
                eq_test = re.sub(y, ListOfMetFrom[y], eq_test)

        # Check if the reaction is balanced and not an exchange or sink reaction
        if len(species) > 1 and all_met_have_formula_test == 1 and not test_reaction_balance(eq_test)[0]:
            MB = mass_balance(eq, 'R') # Get the mass balance for the reaction
            # Get the new species and stoichiometric coefficients
            NewSpecies = MB[6]+MB[7] # Species added to substrates and/or products to mass balance
```

NewStoichimotry = MB[0]+MB[1] # Stoichimetry of the reblanced reaction
    # Modify x2 according to NewSpecies and NewStoichimotry
  ## S-GPRs and Location
  for ec in EC:
    new_gpr = getGPR(ec, session) # build a new S-GPR and GPR
    if new_gpr:
      new_locations = getLocation(new_gpr[3], new_gpr[1], new_gpr[2], 1, location_pkl_file, session) # Identify the subcellular location based on the genes in the GPR
      variables[ec].extend(new_gpr) # Add new GPR to the entry in the dictionary varibles with key "ec"
      variables[ec].extend(new_locations) # Add new Subcellular location to the entry in the dictionary varibles with key "ec"
      variables[x.id] = meltGeneList(new_gpr in variables[ec], new_location in variables[ec]) # integrate the all the new_gpr and new_location of a given reaction
  for i in variables[x.id]:
      CSL = ith subcellular location with the corresponding S-GPR/GPR and genes
      if x.id for the ith subcelular location does not exist in the model:
        Build a new reaction with the attibutes in x.id and the subcellular location specific information in CSL
      else:
        Update the information regarding genes and S-GPR/GPR in the existing reaction

E. Sanitize model and store the final model in a SBML file
- Eliminate duplicate reactions
- Eliminate unconnected metabolites
- Rebalance metabolic reaction
- write_sbml_model(sanitized_model, "Output_model.xml") # Writte the sbml model:

3. Pseudo Code for "Building model" process

# A. Build a metabolic network based on a list of pathways, add reactions, metabolites, ensure network consistecy, curate annotation and ensure mass balance
Path = List of metabolic pathways containing pathway name and id (i.e. KEGG)
i = 0
while i < len(Path) – 1:
  # Extarct pathway information
  PathID = split_path_id(Path[i]) # pathway id
  PathName = split_path_name(Path[i]) # pathway name
  PathURL = build_path_url(PathID) # patwhway url
  PathReferer = build_path_referer(PathID)
  PathList[PathID] = create_pathway_object(PathURL, time, PathID, PathReferer, PathName) # Pathway object gathering information from different databases
  PathNameRxn[PathName] = "" # empty dictionary for the reaction in the pathway
  j = 0
  while j < len(PathList[PathID].Reactions()):
    RxnID = get_reaction_id(j, PathList[PathID]) # Reaction identifier in the database that will be used in the model
    if not_rxn_id_in_ident_or_equiv(RxnID): # add reaction if reaction is not previously annotated
      RxnIdent = add_rxn_id_to_ident(RxnID, RxnIdent) # add reaction ids

```
            RxnURL, RxnTermDyn = get_rxn_url_and_term_dyn(j, PathList[PathID]) # reaction url and
reversibility
            RxnList[RxnID] = create_reaction_object(RxnURL, time, RxnID, PathName, RxnTermDyn) #
Reaction object gathering information from different databases
            RxnCmp = get_rxn_compounds(RxnID, RxnList) # identify reaction's substrates and products
            c = 0
            while c < len(RxnCmp):
                CompID = RxnCmp[c]
                if not_comp_id_in_ident_or_equiv(CompID): # add metabolite is not previously annotated
                    MetIdent = add_comp_id_to_ident(CompID, MetIdent)
                    CompURL = build_comp_url(CompID)
                    MetList[CompID] = create_compound_object(CompURL, CompID, time, EF,
specialCompounds) # Metabolite object gathering information from different databases
                c = c + 1
            # Mass balance reaction
            ithRxn = RxnList[RxnID]
            eq, mb_test = get_eq_and_mb_test(ithRxn, MetList) # test if reaction is umbalanced
            LibIni = wrap_rxn_subs_prod_param(ithRxn, MetList) # extract information from the reaction
object to be lately reintroduced together with the balanced reaction
            if mb_test != 0: # mass balace the reaction in case of unbalance
                IthRxnMB = mass_balance(eq, RxnID) # Mass balance the metabolic reaction
                # If an additional metabolite has to be added to mass balance the metabolic reaction check if
it/they is/are already added to the model, and if not add it/them
                if IthRxnMB[4]:
                    for x in IthRxnMB[4]:
                        if not extra_compound[x[0]] in MetIdent and not extra_compound[x[0]] in MetEquiv:
                            MetIdent = add_extra_comp_id_to_ident(x[0], MetIdent)
                            extra_url = build_extra_comp_url(extra_compound[x[0]])
                            MetList[extra_compound[x[0]]] = create_compound_object(extra_url,
extra_compound[x[0]], time, EF, specialCompounds) # Metabolite object gathering information from
different databases
                RxnList[RxnID] = update_rxn_substrate_product_and_kinetics(ithRxn, LibIni, ithRxnMB,
MetList, MetEquiv) # update the reaction object with the rebalanced reaction
                update_rxn_list(RxnID, RxnList) # add updated information to the list of reactions
        j = j + 1
i = i + 1

# B: Gene annotation and build S-GPRs and GPRs
GeneList = {} # Initialize an empty dictionary to store gene information
GeneIdent = [] # Initialize an empty list to store gene identifiers
g = 0
# Loop through each gene-protein-reaction (GPR) association in the list
while g < len(GPRList):
    # Check if the gene identifier is already in the dictionary and has subcellular location information
    if GPRIdent[g] in GPRList.keys() and GPRList[GPRIdent[g]].GprSubcell():
        # Extract the gene names from the subcellular location information
        gene_matches = re.findall(
            "([A-Za-z0-9\-]+)",
            GPRList[GPRIdent[g]].GprSubcell()[1].replace("and", "").replace("or", ""),
```

```
    )
    # Loop through each gene name in the list
    z = 0
    while z < len(gene_matches):
        # If the gene name is not already in the list of gene identifiers, add it and its information to the dictionary
        if not gene_matches[z] in GeneIdent:
            GeneIdent = GeneIdent + [gene_matches[z]]
            GeneList[gene_matches[z]] = gene(gene_matches[z], EnsblDB) # Gene object gathering information from different databases
        z = z + 1
  g = g + 1

# C: Identify sub-cellular locations
# Remove any non-alphanumeric characters from the compartment name and get a master list of IDs
CSL2 = re.sub(r'[^A-Za-z0-9 ]+', '', CSL)
ModMaster = list(set(LipidMasterlistOfID + listOfID))
# Assign the ID to the CSL if it already exists in the CSL_ID dictionary, otherwise generate a new ID
if CSL_ID.get(CSL):
  ID = CSL_ID.get(CSL)
elif len(re.sub(" $", "", re.sub("^ ", "", CSL)).split(" ")) > 1:
  ID = (CSL2.split(" ")[0][0] + CSL2.split(" ")[1][0]).lower().replace(" ", "")
else:
  if len(CSL.split(" ")) > 1:
    ID = CSL2[0:3].lower().replace(" ", "")
  else:
    ID = CSL2[0:2].lower().replace(" ", "")
# If the generated ID already exists in the master list, append a number to the end to make it unique
if not CSL_ID.get(CSL) and ID in ModMaster:
  r = re.compile(ID)
  ID = ID + str(len(list(filter(r.match, ModMaster))) + 1)
# Assign the ID to the compartment in the LocVar dictionary
LocVar[CSL] = ""
LocVar[CSL] += ID
# Append the ID to the list of IDs and the master list of Lipid IDs
listOfID.append(ID)
LipidMasterlistOfID.append(ID)

# D: Gather all the information collected in the previous steps and write it in a model in SBML format
model = cobra_reconstruction(
  ModName, ModID, MetList_CL, RxnList_CL, GeneList, PathNameRxn, LocVar, MetEquiv, MetList
)
cobra.io.write_sbml_model(model, Output)
```

4. Pseudo Code for "/merge_metabolic_netowrks_and_network_consistency/" process

```
# A: Load input data
```

```
network_1 = read_sbml_model(GEM1) # target network (i.e. network from GEM)
network_2 = read_sbml_model(GEM2) # source network (i.e. netwokr from DBs)
# B: Merge metabolites
network_3 = network_metabolites_merge(network_1, network_2)
# C: Merge genes
network_3_genes = network_genes_merge(network_3_metabolites, network_2)
# D: Merge reactions and test network consistency
network_3_reactions = network_reactions_merge(network_3_genes_model, network_2)
# E: Sanitize model
new_network_4_metabolites = delete_isolated_metabolites(network_3_reactions)
new_network_4_reactions = delete_not_used_reactions(new_network_4_metabolites)
new_network_4_genes = delete_not_used_genes(new_network_4_reactions)
# F: Write output model in a file
write_sbml_model(new_network_4_genes, Output)
```
