# Supplementary material for: A Protocol for the Automatic Construction of Highly Curated Genome-Scale Models of Human Metabolism
Source: Bioengineering (Basel). 2023 May 10;10(5):576. doi: 10.3390/bioengineering10050576 (PMC10215578; doi:10.3390/bioengineering10050576)
Supplement: Supplementary file 1 [file bioengineering-10-00576-s001.zip › Marin de Mas et al-Supplementary Material file S4.zip!/Important note on MEMOTE analysis of THG model.doc]

Important note for the MEMOTE analysis of THG:

In the presented protocol, in the merging step (Steps 4, 5 and 6 Figure 1 in the main text) the stoichiometric consistency of the metabolic network is tested for each new reaction that is added to the THG_β2_ from the human database. The network stoichiometric consistency is later used by MEMOTE as one of the parameters to evaluate the quality of the model. More specifically this task is performed by the function “consistency.check_stoichiometric_consistency”, defined in the MEMOTE protocol. In some particular cases this function can present numerical instability, providing a false negative when the stoichimetric consistency of a network is assessed. In other words, a given metabolic model can be wrongly defined as stoichiometrically inconsistent. This numerical instability can also affect the mass balance analysis of the metabolic reactions.

This error raised when assessing THG model, which affected the overall score of the model in the MEMOTE analysis. In order correctly determine the stoichimetric consistency of THG model the “findStoichConsistentSubset” function was used. This function is defined in the COBRA3.0 Toolbox for Matlab [1] and overcomes the limitations presented by the function implemented by default in MEMOTE. To calculate the number of mass balanced reactions in the THG model, the mass_balance function developed in this work was used

The function “findStoichConsistentSubset” determined that THG model was stoichimetrically consistent and the mass_balance function identified 89 imbalanced reactions.

Finally the final score of the MEMOTE analysis for THG model was recalculated accordingly based on MEMOTE documentation [2]. This affected the consistency test, it was initially scored with 40 and the recalculation provided a score of 91 and the new overall score was 81.

References:

1. Heirendt, L., Arreckx, S., Pfau, T. *et al.* Creation and analysis of biochemical constraint-based models using the COBRA Toolbox v.3.0. *Nat Protoc* 14, 639–702 (2019).

2. Lieven C, Beber ME, Olivier BG, Bergmann FT, Ataman M, Babaei P, Bartell JA, Blank LM, Chauhan S, Correia K, Diener C, Dräger A, Ebert BE, Edirisinghe JN, Faria JP, Feist AM, Fengos G, Fleming RMT, García-Jiménez B, Hatzimanikatis V, van Helvoirt W, Henry CS, Hermjakob H, Herrgård MJ, Kaafarani A, Kim HU, King Z, Klamt S, Klipp E, Koehorst JJ, König M, Lakshmanan M, Lee DY, Lee SY, Lee S, Lewis NE, Liu F, Ma H, Machado D, Mahadevan R, Maia P, Mardinoglu A, Medlock GL, Monk JM, Nielsen J, Nielsen LK, Nogales J, Nookaew I, Palsson BO, Papin JA, Patil KR, Poolman M, Price ND, Resendis-Antonio O, Richelle A, Rocha I, Sánchez BJ, Schaap PJ, Malik Sheriff RS, Shoaie S, Sonnenschein N, Teusink B, Vilaça P, Vik JO, Wodke JAH, Xavier JC, Yuan Q, Zakhartsev M, Zhang C. MEMOTE for standardized genome-scale metabolic model testing. *Nat Biotechnol*. **2020**, *38(3)*, 272-276.
